# Heterologous expression, purification and function of the extracellular domain of human RANK

**DOI:** 10.1186/s12896-017-0405-y

**Published:** 2017-12-04

**Authors:** Yilei Wei, Yu Zhan, Pengfei Chen, Zhi Liu, Haohao Zhang, Dandan Liu, Jie Zhang, Min Yu, Wei Mo, Jun Zhang, Xiaoren Zhang

**Affiliations:** 1 Department of Blood Transfusion, The First Affiliated Hospital of Bengbu Medical College, Bengbu, China; 2 Key Laboratory of Stem Cell Biology, Shanghai Institutes for Biological Sciences, University of Chinese Academy of Sciences, Chinese Academy of Sciences, Shanghai, 200031 China; 3 Collaborative Innovation Center of System Biomedicine, Shanghai Jiao Tong University School of Medicine, Shanghai, 200240 China; 4 Key Laboratory of Metabolism and Molecular Medicine, Ministry of Education, Fudan University, Shanghai, China

**Keywords:** RANK, *Pichia pastoris*, Protein purification, Bone diseases, Colorectal cancer

## Abstract

**Background:**

Receptor activator of NF-κB ligand (RANKL)/RANK signaling essentially functions within the skeletal system, particularly participating in osteoclastogenesis and bone resorption. In addition, this signaling pathway has also been shown to influence tumor progression as well as the development and function of the immune system. Therefore, blocking the interaction between RANKL and RANK is a new therapeutic approach to prevent bone-related diseases and cancer.

**Results:**

The coding sequence encoding the extracellular domain of human RANK (RANK-N) was codon optimized for *Pichia pastoris* and cloned into the pPIC9K vector, and the recombinant plasmid was then transformed into *P. pastoris*. The expression of RANK-N protein was confirmed using SDS-PAGE with Coomassie Brilliant Blue stain and western blotting. Recombinant RANK-N protein was purified by a multistep process including ultrafiltration (UF), Sephadex G-50 size-exclusion chromatography and Q-Sepharose Fast Flow ion exchange chromatography, which resulted in a purity >95%. We found that the RANK-N protein can block RANKL-RANK signaling both in vitro and in vivo. Furthermore, using a patient-derived xenograft of human colon cancer, we found that the recombinant RANK-N protein can inhibit the growth of colorectal cancer.

**Conclusions:**

The results show that a simple system to express and purify functional RANK-N protein has been developed. This work has thus laid a foundation for further research and clinical applications of RANK-N protein in treating bone-related diseases or even colorectal cancer.

**Electronic supplementary material:**

The online version of this article (10.1186/s12896-017-0405-y) contains supplementary material, which is available to authorized users.

## Background

RANK, the signaling receptor for RANKL, is a 616-amino acid homotrimeric transmembrane protein which belongs to the tumor necrosis factor (TNF) receptor family. RANK is constitutively expressed in multiple organs, including skeletal muscle, thymus and skin. And it is strongly induced on osteoclast precursors by macrophage colony-stimulating factor (M-CSF), suggesting that it has a number of important physiological functions [1–5].

RANKL/RANK signaling is initiated by the recruitment of adaptor and docking proteins, most importantly TNF receptor-associated factors (TRAFs) [6], which activate downstream signaling pathways including nuclear factor-κB (NF-κB) and MAPK pathway such as c-JNK, p38, and the extracellular signal-regulated kinases (ERK) [7].

The RANKL/RANK system was originally described by different groups as being critical for T-cell activation, dendritic cell survival [1, 8], and bone homeostasis by the regulation of the differentiation of osteoclasts (OCs) [9, 10]. Recent studies have demonstrated that the RANKL/RANK system also plays an critical role in the immune system, including in lymphoid organogenesis, T cell activation and differentiation, dendritic cell survival, and central tolerance induction [11–13].

In this study, RANK-N, a 185-amino acid soluble form of the extracellular domain of RANK, was designed to disrupt the RANKL-RANK signaling pathway. *Pichia pastoris*, currently named Komagataella phaffii, a yeast that is widely used as an high efficiency and inducible protein expression system, was used to efficiently express RNAK-N protein. RANK-N, induced by methanol, was secreted into the culture medium and purified by a multistep process including ultrafiltration (UF), Sephadex G-50 size-exclusion chromatography and Q-Sepharose Fast Flow ion exchange chromatography. We also showed that RANK-N interacted with RANKL, suppressed the downstream signaling by RANK activation in vitro, and inhibited tumor growth in a patient-derived xenograft (PDX) model in vivo. Thus, the RANK-N protein we obtained may be useful for the potential application in treatment of bone-related diseases or even colorectal cancer.

## Methods

### Mice

Balb/c nude mice and all the mice used were purchased from the Shanghai Laboratory Animal Center, Chinese Academy of Sciences (Shanghai, China). All mice were housed and maintained in specific-pathogens-free conditions at the Institute of Health Sciences. All animal experiments were approved by the Institutional Biomedical Research Ethics Committee of the Shanghai Institutes for Biological Sciences (SIBS), Chinese Academy of Sciences (CAS) and performed in compliance with the Guide for the Care and Use of Laboratory Animals [14].

### Strains and plasmids

The plasmid pPIC9K and the yeast cell strain GS115 were obtained from the Key Laboratory of Molecular Medicine of Fudan University [15]. The *E. coli* strain DH5α was obtained from TIANGEN Biotech Co., Ltd. (Beijing).

Yeast nitrogen base (with or without amino acids) was purchased from Sigma. Q-Sepharose-FF and Sephadex G-50 were purchased from GE Healthcare.

### Construction of expression vector pPIC9K/RANK-N

The gene fragment encoding RANK-N (amino acids 29 to 213) is a part of the *RANK* gene (85–639 bp). A version of *RANK-N* gene fragment encoding RANK-N was codon optimized (*Pichia pastoris*)*,* synthesized and cloned into a pUC57 plasmid (Additional file 1). The procedure was performed in Sangon Biotech. The *RANK-N* fragment was then integrated into pPIC9K that had been digested with *Xho*I and *Not*I (Thermo Scientific). The presence of pPIC9k/*RANK-N* in successful recombinant colonies was confirmed by gene sequencing.

### Transformation and selection of recombinant *P. pastoris* colonies that highly express the target protein

The plasmid pPIC9K/*RANK-N* was cut with restriction enzyme *Sal*I. The digested products were then purified using an EasyPure PCR Purification Kit (Transgene Biotech), electroporated with a MicroPulser™ Electroporator (Bio-Rad) and transformed into the yeast strain GS115. The transformants were cultured on MD plates (2% glucose, 4 × 10^−5^% biotin, and 1.34% YNB) for 2–3 days. We picked up about 1000 colonies for further screening with G418 (Amresco E859-5G). Strains that could grow at high concentrations (2 mg/ml or more) of G418 were stored at −80 °C.

Each clone obtained was then streaked on a new YPD plate to obtain single colonies. Next step, each single colony was inoculated in 250 mL flasks with 50 ml BMGY medium and cultured at 30 °C in a shaker at 220 rpm. When the OD_600_ reached 3–4 (logarithmic phase), we harvested and rinsed the cells. Then rinsed cells were resuspended in 50 mL BMMY and cultured in the same shaker of 220 rpm at 30 °C, and the culture was supplemented with 500 μl methanol to a final concentration of 1% every 12 h. An 80-μl aliquot of the supernatant was collected at every 12 h so that the expression of RANK-N could be determined using SDS-PAGE and western blotting.

### Purification of the RANK-N

After 96 h of induction, the total supernatant from all the flasks (approximately 2 l) was applied into an ultrafiltration system (Merck Millipore, P2PLBCC05) and concentrated to approximately 200 mL in volume. Then, the 200-mL sample was loaded to a Sephadex G-50 size exclusion column which had been preequilibrated with Tris-HCl buffer (20 mM, pH 9.0). The fraction with RANK-N was then applied to a Q-Sepharose-FF column installed in an ÄKTA explorer 100 system at 5 mL/min. The column was washed with Tris-HCl buffer (20 mM, pH 9.0) until the absorbance at 280 nm reached the base level. After that, a linear gradient of washing buffer (1.0 mol/L NaCl in 20 mM Tris-HCl pH 9.0) was pumped to the column to wash the protein off from the column. The protein fractions with RANK-N was harvested, lyophilized and stored at −80 °C.

### Western blot and Coomassie Brilliant Blue staining

SDS-PAGE analysis was carried out according to the standard protocol. Gels were stained in Coomassie Brilliant Blue R-250 solution overnight or for at least 3 h and then destained in destaining buffer. For WB analysis, the proteins in the gel were transferred to a PVDF membrane (Immobilon P, Millipore) using a wet electroblotting system (Bio-Rad) working at 100 V for 50 min in a Tris-glycine running buffer (192 mM glycine and 25 mM Tris). The membrane with protein was then blocked in 5% non-fat milk for 1 h at room temperature (RT), after which it was first immunoblotted with specific antibodies at 4 °C overnight and then with HRP-conjugated secondary antibody for 1 h at Room Temperature. The signals of the bound antibodies were detected using Super Signal West Pico Chemiluminescent Substrate (Pierce) in a LAS4000 camera system (Fujifilm). The specific antibody against RANK-N (sc-7626) was purchased from Santa Cruz Biotechnology.

### Biacore T100 analysis

Surface plasmon resonance experiments were carried out with a Biacore T100 protein interaction analysis system (GE Healthcare, Little Chalfont, UK). RANKL (R&D Systems, 462-TEC-010/CF) protein was covalently coupled to a CM5 sensor chip (GE Healthcare) using an amine coupling kit (GE Healthcare). RANK-N was diluted with PBS-EP + running buffer (3 mM EDTA, 150 mM NaCl, 10 mM HEPES and 0.05% (*v*/v) Surfactant P20) and then injected over the sensor chip at 30 μl min^−1^ at 25 °C (contact time: 210 s, dissociation time: 400 s). After each measurement of a binding response (in resonance units, RU), the sensor chip was regenerated using 10 mM glycine–HCl buffer (pH 2.5). The values of dissociation rate constant (*k*_*d*_), the association rate constant (*k*_*a*_) and equilibrium dissociation constant (*K*_*D*_) were derived by simulating the binding curves with a monovalent interaction model using BIA evaluation software (GE Healthcare).

### Sugar content analysis of the recombinant protein

A total of 10 μg of purified RANK-N was treated with 1 μL PNGase F (NEB, P0704S) and 2 μL of 10% NP40 for 1 h, and then denatured at 100 °C for 10 min according to the manufacturer’s instruction. Finally, the treated protein was analyzed using Coomassie Brilliant Blue staining.

### Inhibition of bone marrow-derived macrophage differentiation into osteoclasts by RANK-N

Bone marrow-derived macrophages were cultured in RPMI-1640 medium containing 10% (vol/vol) FCS, 50 μM 2-mercaptoethanol and 10 ng/ml M-CSF (PeproTech). Then, 1 μg/ml RANKL was added to induce bone marrow-derived macrophages (BMDMs) to differentiate into osteoclasts, and TRAP staining (Sigma) was performed to evaluate the differentiation process.

### PDX tumor model experiment

Colorectal tumors were collected at the time of surgery, washed with phosphate-buffered saline (PBS) and injected subcutaneously into the flanks of 6-week-old athymic mice after being cut into pieces approximately 2 mm in size. Mice were observed daily, and tumors were measured with vernier calipers until the volume of the tumor (V  =  L × 2 W × 0.52 (L = longest diameter, W = shortest diameter)) reached approximately 1000 mm^3^. Tumors were then harvested, minced and reimplanted as described above until stable PDX was established.

The tumor-grafted mice were blindly divided into two groups (4 mice per group), and RANK-N (100 mg/kg) or PBS was injected intraperitoneally (i.p.) every other day for a total of 2 months. Antitumor activity of treatments was evaluated by tumor weight and tumor volume. Protein was extracted from the tumor samples, and further analyzed using western blotting.

### Data analysis and statistics

Statistical analysis was carried out using GraphPad Prism 5.0 software. The data are presented as the mean ± SD and analyzed using unpaired T. test, *p* < 0.05 was considered statistically significant. All experiments in this study were performed at least three times.

## Results

### Construction of the expression system and expression and characterization of RANK-N

The *RANK-N* gene fragment encoding RANK-N, a part of DNA fragment of the *RANK* gene (85–639 bp), was codon optimized for expression in *Pichia pastoris* and then cloned into the expression vector pPIC9K cut with *Xho*I and *Not*I. DNA sequencing confirmed the correct sequence with no frameshift (Additional file 1).

**Fig. 1:**
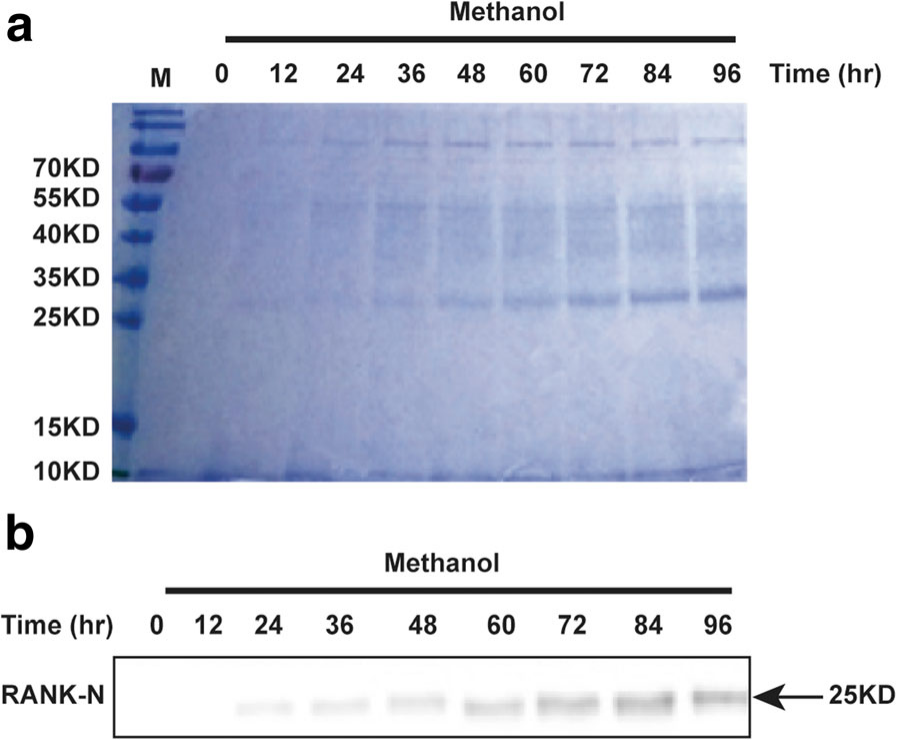
Expression and characterization of recombinant RANK-N. **a** Samples were taken from culture medium of pPIC9K /RANK-N after 0, 12, 24, 36, 48, 60, 72, 84 and 96 h of induction by methanol. The supernatant samples were separated in a 12% (*w*/*v*) SDS-PAGE gel and stained with Coomassie Brilliant Blue R-250. **b** Western blot analysis of RANK-N expression after 0, 12, 24, 36, 48, 60, 72, 84 and 96 h of induction by methanol

Transformation of *P. pastoris* with pPIC9K/*RANK-N* produced 8 transformants with the screening in the presence of a gradually increased concentrations of geneticin. Six and two strains grew on YPD plates with 4 and 6 mg/mL geneticin, respectively. Then all the strains were cultured in BMMY medium to induction of the expression of RANK-N. The supernatant samples, collected at every 12 h, were analyzed after methanol induction for 96 h. Coomassie Brilliant Blue Staining indicated the presence of a band at approximately 28 kDa whose intensity increased and then peaked at 96 h, suggesting that RANK-N might be expressed and secreted into the culture media (the theoretical size of RANK-N is 21 kDa) (Fig. 1a). Furthermore, the western blotting result showed that the band that was detected by Coomassie Brilliant Blue staining was specifically recognized by a RANK specific antibody, demonstrating that the protein secreted by *P. pastoris* was recombinant RANK-N (Fig. 1b).

### Purification of RANK-N

**Fig. 2:**
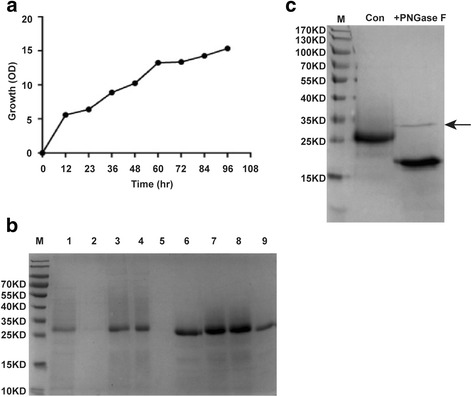
Purification of RANK-N and glycosylation analysis. **a** The growth curve of *Pichia pastoris* in a shake flask at 30 °C. **b** SDS-PAGE analysis of purified RANK-N protein. M: molecular weight marker; Lane 1: Induction by methanol at 96 h; Lane 2: Flow-through of ultrafiltration; Lane 3: Sample after ultrafiltration; Lane 4: Fraction 1 of Sephadex G-50 gel chromatography; Lane 5: Flow-through from Q-Sepharose-FF chromatography; lanes 6–9: Fraction 1 of Q-Sepharose Fast Flow chromatography. **c** Treatment of the protein with PNGase F. Lane 1: molecular weight marker; Lane 2: RANK-N; Lane 3: RANK-N+ PNGase F, arrow indicates PNGase F

Among all the RANK-N expressing strains obtained, strain no. 7 was able to grow on 6 mg/ml geneticin plate and accordingly expressed RANK-N at the highest level, which was chosen for further studies. First it was grown in a 2-l stirred beaker flask for 96 h, and expression was induced with methanol, as described in the Material and Methods. The growth curve of the yeast is shown in Fig. 2a. Then the culture medium was centrifuged, and the supernatant was concentrated to a volume of 200 mL by ultrafiltration (Merck Millipore, P2PLBCC05). The concentrated supernatant was loaded onto a Sephadex G-50 size-exclusion column. The flow-through sample was collected separately and analyzed by SDS-PAGE; Coomassie Brilliant Blue staining showed that the RANK-N protein was in the fraction corresponding to the first ultraviolet absorption peak. Then, the fraction containing RANK-N (160 mL) was pumped onto a Q-Sepharose-FF column, and the targeted protein was obtained at the washing step that used 0.03 M NaCl in washing buffer. The staining results are shown in Fig. 2b. Generally, we obtained about 8.1 mg high purity RANK-N each time.

### Sugar content of the recombinant protein

To determine whether the RANK-N protein was glycosylated the purified protein was treated with PNGase F according to the manufacturer’s protocol. As shown in Fig. 2c, Coomassie blue staining indicated a single 28-kDa band before enzyme treatment and a band of about 21 kDa after PNGase F treatment. This result indicated that the RANK-N obtained was N-glycosylated. The predicted glycosylated sites are at Asn 105 and Asn 174 using NetNGlyc 1.0 Server, and the corrected sites need to be further verified. The band at approximately 35 kDa is the enzyme PNGase F.

### Rank-N and RANKL binding activity

**Fig. 3:**
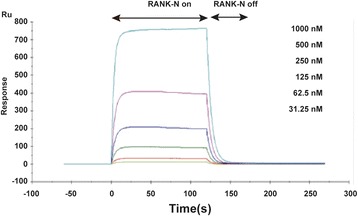
Biacore T100 analysis of RANKL/RANK-N binding. Concentrations of RANK-N (from 32.5 to 1000 nM) were passed over a CM5 chip that was coated with RANKL. The data are plotted as the steady-state response (in RU) vs RANK-N concentration and fit to a rearranged Hill equation, resulting in a K_D_ of 2.62×10^−6^ M

The binding between RANK-N and RANKL was quantitated using Biacore T100 analysis (Fig. 3). RANKL was firstly tightly bound to CM5 chips according to the protocol. RANK-N was diluted in PBS and injected onto the chip that had RANKL bound to its surface. Increasing concentrations of RANK-N (from 32.5 to 1000 nM) were passed over the RANKL-bound chips. The steady-state responses (in RU) were obtained at each RANK-N concentration. These data were fit to the Hill equation to determine the dissociation constant. Our data showed that a value of K_D_ for the interaction was 2.62×10^−6^ M.

### Dose-dependent blocking of noncanonical NF-κB signaling by RANK-N

**Fig. 4:**
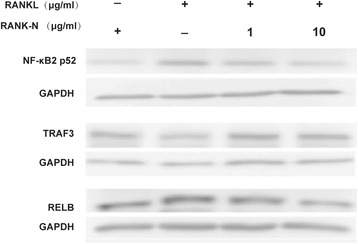
Dose-dependent blocking of noncanonical NF-κB signaling by RANK-N. Western blot analysis of the processing of NF-κB2 p100 into NF-κB2 p52 in RANKL-induced Raw264.7 cells in the presence of different concentrations of RANK-N (1 and 10 μg/mL)

Purified RANK-N was assayed for its inhibitory effect on RANK-activated signaling in Raw264.7 cells because RANKL can lead to RANK-mediated processing of NF-κB2 p100 into NF-κB2 p52, activating a noncanonical NF-κB signaling pathway [16]. As shown in Fig. 4, RANK-N disrupted the processing of NF-κB2 p100 into NF-κB2 p52 that is induced by RANKL in Raw264.7 cells in a dose-dependent manner, proving that RANK-N could disrupt RANKL activated RANK signaling. These data demonstrate that the purified protein was functional in vitro.

### Inhibition of BMDM differentiation by RANK-N

**Fig. 5:**
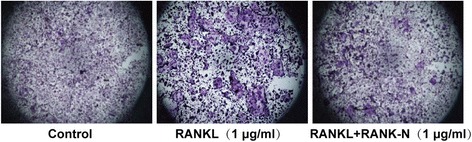
RANK-N inhibition of bone marrow-derived macrophage (BMDM) differentiation. TRAP staining of osteoclasts differentiated from RANKL-induced BMDMs in the presence of RANK-N (1 μg/mL)

As it was previously reported that RANKL can induce the differentiation of BMDMs into osteoclasts, we further evaluated the blocking activity of RANK-N using this model [17]. TRAP staining showed that 1 μg/ml RANK-N indeed inhibited the differentiation of BMDMs into osteoclasts induced by RANKL (Fig. 5).

### Inhibition of tumor growth by RANK

**Fig. 6:**
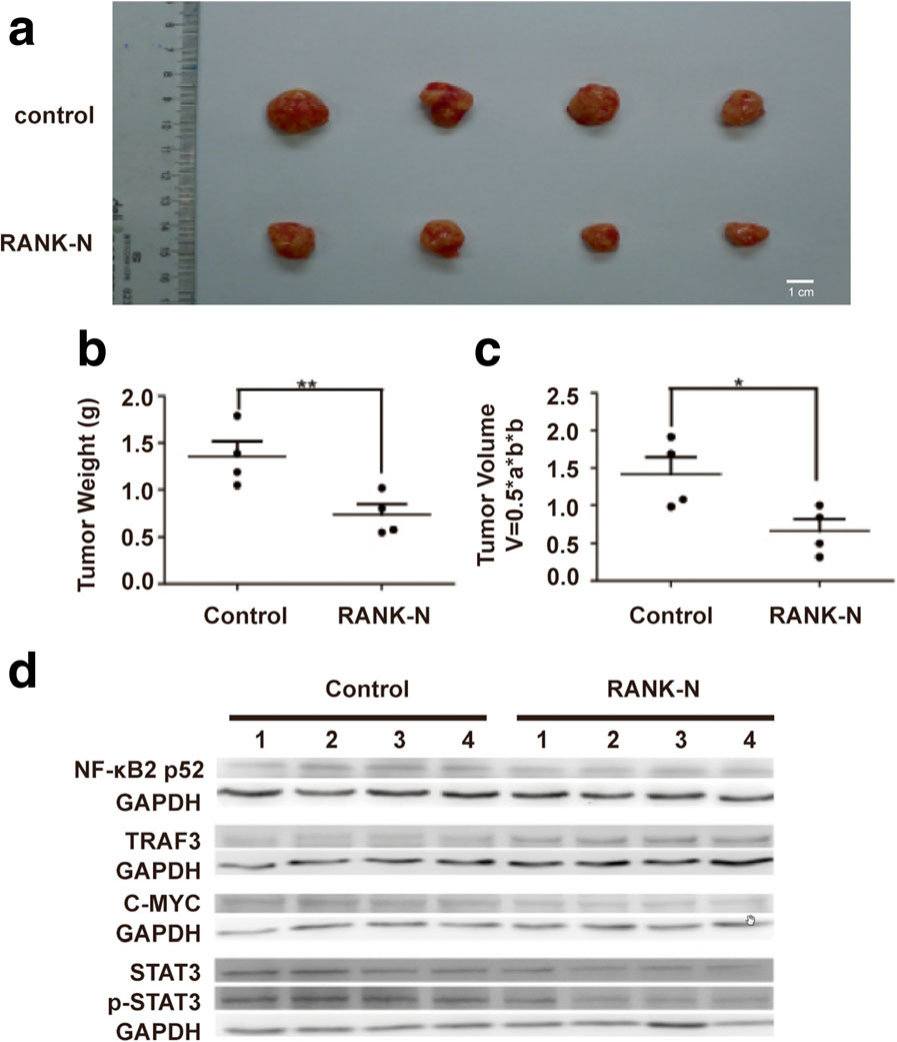
Inhibitory effect of RANK-N on tumor growth in vivo. **a** Tumors were isolated at 6 weeks post-injection. **b**-**c** Tumor weight and volume were measured and calculated. *p < 0.05, **p < 0.01. **d** The levels of NF-κB2 p52, TRAF3, c-Myc, STAT3 and p-STAT3 proteins in xenograft tumors were analyzed by western blotting

To determine whether RANK-N works in vivo*,* we analyzed the antitumor effect of RANK-N in a PDX model by transplanting a tumor that was derived from colorectal cancer patients into athymic nude mice. The mice were randomly divided into 2 groups 7 days after implantation. Mice with tumors were injected with 10 mg/kg RANK-N i.p. every other day for 6 weeks in the RANK-N group and with PBS in the control group. We found that RANK-N significantly inhibited the growth of xenograft tumors (Fig. 6a-c). Moreover, the processing of NF-κB2 p100 to NF-κB2 p52 and the expression of c-Myc, STAT3 and p-STAT3 decreased in the RANK-N group compared with the control group (Fig. 6d), suggesting that RANK-N might inhibit tumor progression by suppressing alternative NF-κB and STAT3 signaling.

## Discussion

Targeting the RANKL-RANK interaction could be useful in clinical applications for treating bone-related disorders and cancer metastasis [13] The strategies to disrupt this interaction include the usage of denosumab, which is an anti-RANKL monoclonal antibody, and OPG-Fc. Denosumab is applied in clinical prevention of SREs (skeletal-related events) in cancer metastasis and fractures in osteoporosis. Clinical trials has revealed that denosumab does not alter the rates of infection or new cancers [18]. Recombinant OPG-Fc (AMGN-0007), competitively binding RANKL with high affinity, was developed and investigated in a clinical trial to treat multiple myeloma and breast cancer bone metastases [19]. However, OPG-Fc had a short half-life and no specificity for RANKL because OPG can also bind the TNF-related apoptosis-inducing ligand (TRAIL) [20].

Given the importance of disrupting RANKL-RANK pathway in clinical applications, we generated an RANK-N *P. pastoris* expression and purification system. We utilized a multistep purification method including ultrafiltration (UF), Sephadex G-50 size-exclusion chromatography and Q-Sepharose Fast Flow ion exchange chromatography to purify recombinant RANK-N from the culture medium. The purity of the protein we obtained was more than 95%. The recombinant RANK-N protein interacts with RANKL from R&D System with a K_D_ value of 2.62×10^−6^ M, which is similar to or higher than those reported by other groups [21, 22]. We may further optimize our system to increase the binding activity of RANK-N through mutating glycoslation sites of RANK-N.

The purified RANK-N disrupted RANK-activated noncanonical NF-κB signaling and BMDM differentiation into osteoclasts in vitro. The experiment in vivo with tumor PDX model showed that purified RANK-N could inhibit tumor growth in vivo; however, the role and underlying mechanism of the RANKL/RANK pathway in cancer need to be further investigated.

## Conclusions

This work reports the successful generation of an efficient expression and purification system that expresses, in *P. pastoris*, large amounts of recombinant RANK-N that can disrupt the RANKL-RANK interaction. The protein fragment that we have acquired may be used in research or even in treating SREs.

## Additional file

Additional file 1: DNA sequence of *Pichia pastoris* favored codon optimize of RANK -N(human). (DOCX 12 kb)

## Abbreviations

BMDMs: Bone marrow-derived macrophages
BMGY: Buffered glycerol-complex medium
BMMY: Buffered methanol-complex medium
bp: Base pair
EDTA: Ethylenediaminetetraacetic acid
HEPES: Hydroxyethyl piperazine ethanesulfonic acid
HRP: Horseradish peroxidase
MAPK: Mitogen-associated protein kinases
M-CSF: Macrophage colony-stimulating factor
PDX: Patient-derived xenograft
rpm: Revolutions per minute
SDS-PAGE: SDS polyacrylamide gel electrophoresis
SPR: Surface plasmon resonance
SREs: Skeletal-related events
TNF: Tumor necrosis factor
TNFRSF: Tumor necrosis factor receptor superfamily
TRAP: Tartrate-resistant acid phosphatase
WB: Western blot
YPD: Yeast extract peptone dextrose medium
