# Supplementary material for: Heterologous expression, purification and function of the extracellular domain of human RANK
Source: BMC Biotechnol. 2017 Dec 4;17:87. doi: 10.1186/s12896-017-0405-y (PMC5716252; doi:10.1186/s12896-017-0405-y)
Supplement: Additional file 1: — DNA sequence of Pichia pastoris favored codon optimize of RANK -N(human). (DOCX 12 kb) [file 12896_2017_405_MOESM1_ESM.docx]

**Heterologous Expression, Purification and Function of the Extracellular Domain of Human RANK**

Yilei Wei^1#^, Yu Zhan^2#^, Pengfei Chen^2^, Zhi Liu^2^, Haohao Zhang^2^, Dandan Liu^2^, Jie Zhang^1^, Min Yu^4^, Wei Mo^4^ , Jun Zhang^1*^ and Xiaoren Zhang^2,3,*^

**Additional file**

DNA sequence of *Pichia pastoris* favored codon optimize of RANK -N(human)

Before:

CGCCTCGAGAAAAGACAGATCGCTCCTCCATGTACCAGTGAGAAGCATTATGAGCATCTGGGACGGTGCTGTAACAAATGTGAACCAGGAAAGTACATGTCTTCTAAATGCACTACTACCTCTGACAGTGTATGTCTGCCCTGTGGCCCGGATGAATACTTGGATAGCTGGAATGAAGAAGATAAATGCTTGCTGCATAAAGTTTGTGATACAGGCAAGGCCCTGGTGGCCGTGGTCGCCGGCAACAGCACGACCCCCCGGCGCTGCGCGTGCACGGCTGGGTACCACTGGAGCCAGGACTGCGAGTGCTGCCGCCGCAACACCGAGTGCGCGCCGGGCCTGGGCGCCCAGCACCCGTTGCAGCTCAACAAGGACACAGTGTGCAAACCTTGCCTTGCAGGCTACTTCTCTGATGCCTTTTCCTCCACGGACAAATGCAGACCCTGGACCAACTGTACCTTCCTTGGAAAGAGAGTAGAACATCATGGGACAGAGAAATCCGATGCGGTTTGCAGTTCTTCTCTGCCAGCTAGAAAACCACCAAATGAACCCCATGTTTACTTGCCCTAAGCGGCCGCATTATTAA

After:

CGCCTCGAGAAAAGACAGATTGCTCCTCCATGTACCAGTGAGAAGCATTATGAGCATCTGGGAAGATGCTGTAACAAATGTGAACCAGGAAAGTACATGTCTTCTAAATGCACTACTACCTCTGACAGTGTATGTCTGCCCTGTGGCCCTGATGAATACTTGGATTCATGGAATGAAGAAGATAAATGCTTGCTACATAAAGTTTGTGATACAGGCAAGGCCTTGGTGGCCGTGGTCGCCGGTAACTCAACGACCCCCAGGCGTTGCGCTTGCACGGCTGGGTACCACTGGTCACAGGACTGCGAGTGCTGTCGTCGAAACACTGAGTGTGCTCCTGGTTTAGGTGCACAACACCCTTTGCAACTAAACAAGGACACAGTTTGTAAACCTTGTCTTGCAGGTTATTTCTCTGATGCATTTTCCTCCACTGACAAATGTAGACCCTGGACTAATTGTACTTTTCTTGGAAAGAGAGTCGAACATCACGGTACAGAGAAGTCCGATGCTGTTTGTAGTTCTTCCTTACCAGCTAGAAAGCCACCAAATGAACCTCACGTTTATTTGCCATAAGCGGCCGCATTATTAA
